# The introduction of mesenchymal stromal cells induces different immunological responses in the lungs of healthy and *M*. *tuberculosis* infected mice

**DOI:** 10.1371/journal.pone.0178983

**Published:** 2017-06-08

**Authors:** Tatiana Nenasheva, Alexander Nikolaev, Daniar Diykanov, Anna Sukhanova, Evgenii Tcyganov, Alexander Panteleev, Irina Bocharova, Yana Serdyuk, Leonid Nezlin, Tatiana Radaeva, Nikolai Adrianov, Yuri Rubtsov, Irina Lyadova

**Affiliations:** 1 Central Tuberculosis Research Institute, Moscow, Russia; 2 Department of Biochemistry and Molecular Medicine, Faculty of Fundamental Medicine, M.V. Lomonosov Moscow State University, Moscow, Russia; 3 Koltzov Institute of Developmental Biology, Russian Academy of Sciences, Moscow, Russia; 4 Pirogov Russian National Research Medical University, Moscow, Russia; University of Michigan Health System, UNITED STATES

## Abstract

Mesenchymal stromal cells (MSC) have strong immunomodulatory properties and therefore can be used to control inflammation and tissue damage. It was suggested recently that MSC injections can be used to treat multi-drug resistant tuberculosis (TB). However, MSC trafficking and immunomodulatory effects of MSC injections during *Mycobacterium tuberculosis* (*Mtb*) infection have not been studied. To address this issue we have analyzed MSC distribution in tissues and local immunological effects of MSC injections in *Mtb* infected and uninfected mice. After intravenous injection, MSC accumulated preferentially in the lungs where they were located as cell aggregates in the alveolar walls. Immunological analysis of MSC effects included detection of activated, IFN-γ and IL-4 producing CD4^+^ lymphocytes, the frequency analysis of dendritic cells (CD11c^+^F4/80) and macrophages (CD11c^-^F4/80^+^) located in the lungs, the expression of IA/IE and CD11b molecules by these cells, and evaluation of 23 cytokines/chemokines in lung lysates. In the lungs of uninfected mice, MSC transfer markedly increased the percentage of IFN-γ^+^ CD4^+^ lymphocytes and dendritic cells, elevated levels of IA/IE expression by dendritic cells and macrophages, augmented local production of type 2 cytokines (IL-4, IL-5, IL-10) and chemokines (CCL2, CCL3, CCL4, CCL5, CXCL1), and downregulated type 1 and hematopoietic cytokines (IL-12p70, IFN-γ, IL-3, IL-6, GM-CSF). Compared to uninfected mice, *Mtb* infected mice had statistically higher “background” frequency of activated CD69^+^ and IFN-γ^+^ CD4^+^ lymphocytes and dendritic cells, and higher levels of cytokines in the lungs. The injections of MSC to *Mtb* infected mice did not show statistically significant effects on CD4^+^ lymphocytes, dendritic cells and macrophages, only slightly shifted cytokine profile, and did not change pathogen load or slow down TB progression. Lung section analysis showed that in *Mtb* infected mice, MSC could not be found in the proximity of the inflammatory foci. Thus, in healthy recipients, MSC administration dramatically changed T-cell function and cytokine/chemokine milieu in the lungs, most likely, due to capillary blockade. But, during *Mtb* infection, i.e., in the highly-inflammatory conditions, MSC did not affect T-cell function and the level of inflammation. The findings emphasize the importance of the evaluation of MSC effects locally at the site of their predominant post-injection localization and question MSC usefulness as anti-TB treatment.

## Introduction

Mesenchymal Stromal cells (MSC) are widely considered as therapeutic cell population capable to dampen undesired immune activation in the course of autoimmunity or tissue regeneration. The concept is based on the immune regulatory, mainly immune suppressive, properties of MSC [1–4]. The suppressive activity of MSC towards T cells was first demonstrated by di Nicola and co-authors who showed inhibition of T cell proliferation *in vitro* in the presence of MSC [5]. The finding was supported by later studies. The cells were shown to inhibit maturation and functions of various immune cells, including macrophages, dendritic cells, NK cells, Th1 and Th17 lymphocytes [6–12]. Recent studies have demonstrated that MSC possess rather immunoregulatory than immunosuppressive properties, and may inhibit, sustain or stimulate effector cell functions depending on the microenvironment [1, 2, 4]. Pro-inflammatory conditions activate MSC to produce suppressive mediators and promote their inhibitory properties [13–16]. However, under steady-state and Th2-biased conditions, MSC are less suppressive and may support various effector immune cells [17]. Thus, the microenvironment fine-tunes MSC function, and the cells may exert different effects in various tissues and pathological conditions [18]. The complexity of MSC effects on immune system and limited understanding of MSC trafficking and distribution *in vivo*, restrict their practical potential and points to the necessity to analyze immunoregulatory properties of MSC in target tissue and in appropriate disease model. Of note, a significant part of data documenting MSC suppressive activity were obtained *in vitro* or under a restricted set of pathological conditions (mainly in animal models of autoimmune diseases and organ transplantation).

Recently, several studies have focused on the potential use of MSC for adjunct therapy of multi-drug resistant tuberculosis (TB) [19–21]. Tuberculosis is a chronic infectious disease, which progression is associated with exacerbated inflammation, lung tissue damage and remodeling (i.e., cavitaion, fibrosis, bronchiectasis) [22–25]. It has been proposed that cellular therapy could limit tissue damage and convert unproductive inflammation into effective pathogen-directed immune responses [20, 21]. However, immune modulatory effects of MSC during *Mtb* infection have not been analyzed in details.

In this study we have used a mouse model to analyze MSC trafficking and MSC-mediated modulation of host immune responses during *Mtb* infection and in steady-state conditions. We specifically focused on local MSC effects in the lungs, a predominant site of *Mtb* infection and MSC accumulation following their systemic administration. We demonstrate differential tissue distribution of MSC following different routes of their injection (i.e., i.v. *versus* i.p.) and differential immunomodulatory effects in the lungs under steady-state conditions and during *Mtb* infection. Our results show that MSC can exert significant immunomodulatory effects in uninflamed lungs and have only limited capacity to modulate local immune responses during *Mtb* infection. Thus, caution should be taken when considering MSC therapeutic usefulness, and the potential of MSC for TB treatment needs further investigation.

## Materials and methods

### Mice

C57BL/6YCit (B6) mice (2–3 months old) were used in the experiments. Mice were housed in the Animal Care Facility of the Central Tuberculosis Research Institute (Moscow, Russia) in accordance with the Russian Ministry of Health Guideline no. 755 and the US National Institutes of Health Office of Laboratory Animal Welfare Assurance #A5502-01. Water and food were provided *ad libitum*. All experimental procedures were approved by the CTRI Institutional Animal Care and Use Committee (IACUC protocols №3, 12, 14, 15 approved on 08 September 2014).

### MSC isolation, passaging and harvesting

MSC were isolated from the adipose tissue of adult mice. Adipose tissue was collected, minced and digested in RPMI-1640 medium containing 200 Units/mL collagenase IA and 50 Units/ml DNAse (Sigma, Aldrich, USA, 40 min, 37°C). The cells were washed and cultured in 25T-flasks (Corning, USA) in DMEM high glucose media (Paneco, Russia) supplemented with 10% FCS, 1% L-glutamine, 1% penicillin/streptomycin (Gibco, USA). After the cells had reached the 90–100% confluence, they were passaged using standard trypsinization detachment procedure (0,25% Trypsin-EDTA, Paneco, Russia). After 2–5 passages, MSC were harvested by trypsinization, washed, counted, phenotyped and used for the adoptive transfer.

### Characterization and labeling of MSC

The cells were analyzed for their phenotype starting the stage of the 2^nd^ passage. 5x10^5^ cells were pre-treated with anti-CD16/32 mAbs (clone 93, eBioscience, San Diego, CA) and stained with PE-Cy7-anti-CD45 (clone 30-F11), PerCP-Cy5.5-anti-Sca1 (clone D7), APC-anti-CD105 (clone MJ7/18), BV510-anti-CD90.2- (clone 53–2.1) and Pacific blue-anti-CD73 (clone Ty/11.8) (all BioLegend, San Diego, CA) or PE-anti-CD73 (clone Ty/23, BD Bioscience, USA) mAbs. After being washed, the cells were analyzed by flow cytometry using BD FACS Canto II instrument (BD Bioscience, USA) and FlowJo (Tree Star, Ashland, OR) software. At the moment of cell transfer MSCs were CD45^-^ (93–97%), Sca1^+^CD90^+^ (90–98%), CD105^+^ (50–57%) and largely negative for CD73 (90–99%), i.e. had phenotype close to that described by Choi and coauthors [26]. To analyze MSC distribution and control their transfer, in some experiments before the transfer MSC were labeled with CFSE (Biolegend, San Diego, CA) or PKH26. CFSE labeling was performed by incubating cells in 2.5uM CFSE (1x10^6^ cells/mL, 8 min at room temperature in the dark). The cells were then washed in PBS-10%FCS and resuspended in PBS. Labeling with PKH26 was done according to the manufacturer recommendations (Sigma, Aldrich, USA). Briefly, cells were resuspended in the Diluent C (2x10^6^ cells per 100 μL), mixed with 100 μl PKH26 (final concentration 2x10^–6^M), incubated for 2 min and washed. The efficacy of CFSE and PKH26 staining was controlled by flow cytometry.

### Study design

To analyze MSC trafficking, mice were challenged with *Mtb* (2.2x10^3^ CFU given intratracheally) or left uninfected. MSC were labeled with CFSE and transferred i.p. or i.v (4x10^5^ cells/mouse). CFSE^+^ cells were examined in the lungs, spleen, liver, lymph nodes, bone marrow and blood 3 hours (“day 0”) and 1–12 days post transfer. Because different organs differ by their cellularity, the results were presented as the percent of CFSE^+^ cells out of all cells in the organ (frequency) and out of all injected cells (proportion).

For immunological analyses, mice were infected aerogenically with 600 CFUs/mouse of mid-log-phase *M*. *tuberculosis* (*Mtb*), strain H37Rv Pasteur, using an Inhalation Exposure System (Glas-Col, Terre Haute, IN). Mice were transferred with MSC on days 46–55 post-infection (two i.v. injections of 4x10^5^ MSC with 3 day interval between the injections). Experiments were performed 3 days after the second transfer. Lungs, spleen and blood were collected. Lungs were perfused with 0,02% EDTA-PBS to wash blood vessels and used for: (i) the determination of mycobacterial load (upper right lobe), (ii) cytokine/chemokine content (upper right lobe), (iii) flow cytometry analysis (low and middle right lobes). In some experiments left lobe of the lungs was used for histological analysis. Mycobacterial loads were determined by plating homogenates of spleen and the upper right lobe of the lungs on Dubos agar [27]. Lung tissue pathology was evaluated by preparing serial 6- to 8-mm sections of the left lobe of the lung. The sections were stained with haematoxylin-eosin and analyzed using an Axioskop 40 microscope (magnification 2.56 and 406) equipped with AxioCam MRc 5 camera (Carl Zeiss, Germany). The area of the infiltrated lung tissue was determined using AxioVs 40 software. The percentage of lung tissue affected by TB was calculated as: infiltrated lung tissue area (mm^2^)/(total lung tissue area (mm^2^)x100%. In all experiments, the progression of the infection was monitored by weighting mice before the challenge, and then every 3–7 days until the day of experiment.

To test the “specificity” of MSC effects, in some experiments unrelated large cells, NIH/3T3 (ATCC® CRL-1658™) mouse emryo fibroblasts were used. The cells were grown and transferred to recipient mice exactly as this was done for MSC. The lungs of recipient mice were obtained 3 days after the second cell transfer and the lysates of the upper right lobe were used in multiplex assay.

### Flow cytometry analyses

Suspensions of lung and liver cells were obtained using an enzyme digestion method. Briefly, perfused lungs were cut and digested in RPMI-1640 medium containing 200 Units/mL collagenase IA and 50 Units/mL DNase (Sigma Aldrich) [28]. Livers were cut and enzymatically digested. Suspensions of spleen and lymph node cells were prepared by mild homogenization. Bone marrow (BM) cells were obtained by flushing tibia and femurs. Blood was collected in heparin-treated tubes, lysed, stained and fixed in BD FACS Lysing solution. Cells were treated with anti-CD16/CD32 mAbs (eBioscience, San Diego, CA) and stained with different combinations of the following Abs: FITC-anti-CD62L (cloneMEL-14), PerCP-anti-CD4 (clone RM 4–5), APC-anti-CD69 (clone H1.2F3); APC-anti-CD25 (clone PC61), BV421-anti-CD127 (clone A7R34, all Biolegend, USA San Diego, CA); PerCP-anti-Ly6G/Ly6C(GR1) (clone RB6-8C5); FITC-anti-F4/80 (clone BM8), APC-anti-CD11b (clone M11/70), BV421-anti-IA/IE (clone M5/114.15.2), BV510-anti-CD11c (clone N418, all Biolegend, USA San Diego, CA). To quantify IFN-γ and IL-4 producing cells, lung cells (1x10^6^ cells) were cultured in the presence of *Mtb* sonicate (10 μg/ml) [29]. After 3 h of incubation Golgi Plug (BD Bioscience) was added, cells were cultured for additional 12 h and harvested. The cells were treated with FITC-anti-CD62L, PE-anti-CD27, PerCP-anti-CD4 mAbs, permeabilized (BD FACS Permeabilising solution 2, BD Bioscience, USA) and treated with APC-anti-INF-γ (clone XMG1.2, Biolegend, San Diego, CA) or FITC-anti-IL-4 (clone 11B11), Biolegend). For Foxp3, cells were surface stained and treated with PE-anti-Foxp3 antibodies (clone fjk-16s eBioscience, San Diego, CA) using Foxp3/Transcription Factor Staining Buffer Set and the manufacturer protocol (eBioscience, San Diego, CA).

### Fluorescence and confocal microscopy

To examine the localization of i.v. transferred MSC within the lung tissue, MSC were labeled with PKH26. Lungs of mice transferred with MSC were fixed in freshly prepared 4% formaldehyde in 0.01M phosphate buffered saline (PBS, pH 7.8) overnight at 4°C, washed in PBS, cryoprotected in 30% sucrose in PBS, embedded in Jung tissue-freezing medium, and cut into 30–40 μm-thick slices using cryostat CM 1510–1 (Leica, Germany).

The free-floating sections were rinsed in PBS, counterstained with ActinGreen 488 kit and 5μM Hoechst 33342 (both from Life Technologies, Carlsbad, CA), cleared in TDE (Sigma-Aldrich, MO) and mounted on glass slides in TDE. The preparations were examined in epifluorescence microscope Biorevo BZ-9000 (Keyence, Japan) and laser scanning microscope TCS SPE (Leica, Germany) equipped with appropriate excitation lines and filter sets. Images were processed with BZ-II Analyzer (Keyence) and Imaris (Bitplane, Switzerland), assembled into plates and labelled using Photoshop CC (Adobe, CA).

### *In vitro* mixed cultures

CD4^**+**^ cells were isolated from suspensions of splenocytes using Dynabeads Untouched Mouse CD4 Cells Kit (Thermo) according to manufacturer’s protocol. Cells were counted and plated in RPMI/10% FCS at density 10^7^ cells/ml to 24 well plate covered with anti-mouse CD3 monoclonal antibodies (clone 37.81, Biolegend). Cells were collected after 48 hours of incubation (37^°^C, 5%CO_2_), counted and plated in 24 well plate with MSC at MSC:T cell ratio 1:10 for 48 hours (37^°^C, 5%CO_2_). T cells were collected, counted and stained with fluorophore-conjugated monoclonal antibodies to evaluate surface markers as mentioned above. Stained cells were analysed by flow cytometry.

### Multiplex assay

Cytokines were evaluated in plasma and lung cell homogenates using 23-plex mouse Cytokine Panel (BioRad, Hercules, California, USA) and Bio-plex MAGPIX Multiplex reader (BioRad Hercules, California, USA). Lung homogenates were prepared from the upper right lobe in 1 ml PBS. Plasma and lung homogenates were stored at -80^°^C until the day of analysis. All procedures were done in accordance with the manufacturer’s recommendations.

### Statistical analysis

Data are shown as mean ± SEM. Differences between the means were analyzed using the non-parametric Mann-Whitney test (GraphPad Software Inc., San Diego, CA). Differences were considered significant if p<0.05.

## Results

### MSC accumulate preferentially in the lungs following adoptive transfer

Identity of MSC obtained from the adipose tissue was examined after several passages by staining cells with antibodies against MSC surface markers and markers of cells of hematopoietic origin. Phenotypic analysis showed that MSC cultures had no significant hematopoietic cell contamination and had phenotype similar to MSC surface phenotype described elsewhere, i.e. expressed Sca-1, CD90, CD105 (see Materials and Methods and [26]). Prior to the analysis of immunological activity of MSC *in vivo*, we characterized immunomodulatory activity of MSC *in vitro*. MSC were cultured with CD4^+^ cells isolated from the spleen of uninfected (TB^-^) or *Mtb* infected (TB^+^) mice and stimulated *in vitro* with plate-bound anti-CD3 antibodies. MSC were capable of inhibiting CD4^+^ cell activation (according to the decreased expression of CD69 and increased expression of CD62L on CD4^+^ cells in the presence of MSC, S1 Fig), i.e. exhibited characteristic immunosuppressive functions described in other studies [30–32].

**Fig 1:**
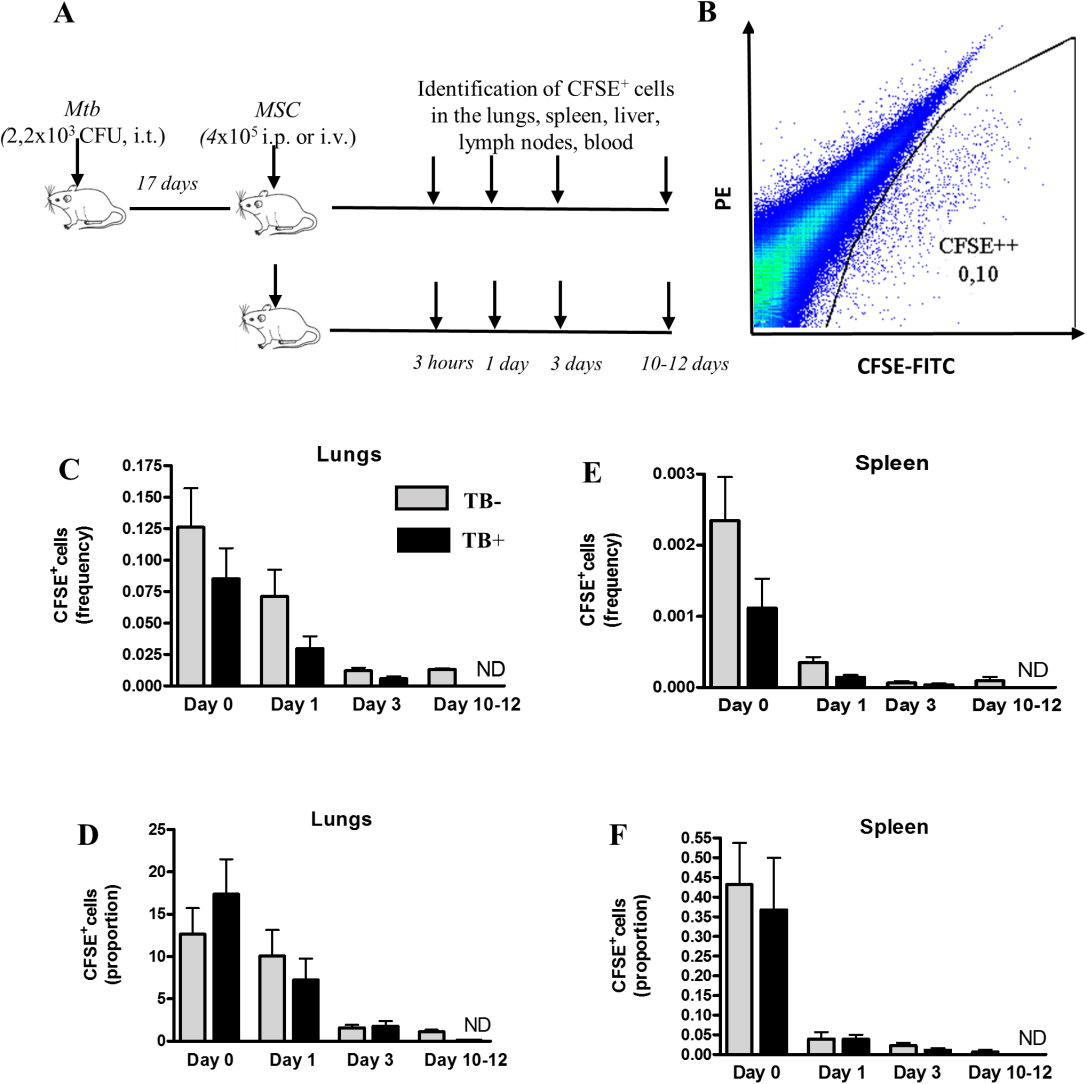
Following intravenous injection, MSC accumulate preferentially in the lungs. MSC were labeled with CFSE and injected i.v. into uninfected and *Mtb* infected mice. Blood, lung, spleen, liver, lymph node and bone marrow cells were prepared 3 hours (day 0) and 1, 3 and 10–12 days post-transfer. CFSE^+^ cells were identified by flow cytometry. A, The scheme of experiments. B, Identification of CFSE^+^ MSC in the lungs of recipient mice (example of flow cytometry data). C,E, Percent of CFSE^+^ cells out of all cells (frequency) in the lungs (C) or spleen (E). D, F, Percent of CFSE^+^ cells out of all injected cells (proportion) found in the lungs (D) or spleen (F). Summarized data of 7 (days 0 and 1) and 2 (days 3 and 10–12) independent experiments (n = 6–19 per time-point).

Next, we analyzed how MSC distributed between different organs of mice after adoptive transfer. CFSE-labeled MSC were injected i.v. or i.p. (4x10^5^ cells/mouse) into TB^-^ or TB^+^ mice. The presence of CFSE^+^ cells was assessed in the lungs, liver, spleen, lymph nodes, blood and bone marrow of recipient mice at different time points (Fig 1A and 1B).

Following i.v. transfer, CFSE^+^ cells accumulated preferentially in the lungs. Three hours post transfer, CFSE^+^ cells made up 0.13±0.10% of total lung cells (frequency). In other words, 12.63±10.24% of injected cells (proportion) were found in the lungs (Fig 1C and 1D). The population of CFSE^+^ cells in the lungs gradually declined by days 10–12. In other organs the frequencies and the proportions of CFSE^+^ cells were significantly lower compared to the lungs at day 0 (30–400 fold, p<0.0001) and were barely detectable at later time-points (Fig 1E and 1F and Table 1).

**Table 1:** Differential distribution of MSC following their i.v. and i.p. transfer.

Organs	MSC transferred i.v.		MSC transferred i.p.	
	Frequency	Proportion	Frequency	Proportion
Uninfected recipients				
Lungs	0.07±0.09	**10.05±13,14**	<0.001	0
Spleen	<0.001	0.040±0,077	**0.004±0.004**	**0.13±0.14**
Liver	<0.001	0.016±0,014	**0.004±0.004**	**0.21±0.45**
Lymph nodes	<0.001	0.006±0,017	<0.001	0
BM	<0.001	<0.001	<0.001	<0.001
Blood	0.001±0.002	0.003±0.003	<0.001	0
*Mtb* infected recipients				
Lungs	0,03±0,04	**7,25±10,95**	<0,001	0
Spleen	<0,001	0,015±0,064	<0,001	0
Liver	<0,001	0,003±0,006	<0,001	0
Lymph nodes	<0,001	0,001±0,002	<0,001	0
BM	<0,001	0,047±0,096	<0,001	0
Blood	0.001±0.002	0.004±0.006	0	0

Uninfected and *Mtb* infected mice were transferred with CFSE labeled MSC as described in the legend to Fig 1. CFSE^+^ cells were identified by flow cytometry, the percentages of CFSE^+^ cells out of all cells in the organ (frequency) were determined and the percentages of CFSE^+^ cells out all injected cells (proportion) were calculated. Shown are data obtained on day 1.

Following i.p. injection, CFSE^+^ cells accumulated almost exclusively in spleen and liver (Table 1). However, even in these organs the frequencies and the proportions of CFSE^+^ cells were significantly lower compared to the frequencies and the proportions of CFSE^+^ cells found in the lungs following i.v. transfer (p<0.001). Starting day 5 post i.p. transfer, CFSE^+^ cells could not been detected in any organ (data not shown).

The pattern of MSC tissue distribution was generally similar in TB^-^ and TB^+^ mice (Fig 1, Table 1 and data not shown).

Overall, MSC distribution depended on the route of their injection. Following i.v. administration, MSC accumulated preferentially in the lungs, which corresponds well to other’s data [33–35]. By day 10 post-transfer, the cells largely disappeared from all the organs.

### MSC differentially affect T cell, macrophage and dendritic cell populations in *Mtb* infected and uninfected mice

Lung tissue represents the main focus of *Mtb* infection. Preferential accumulation of systemically transferred MSC in the lung tissue along with the effects, which pro-inflammatory conditions exert on MSC activity [1–4, 13, 14], raised a question on whether MSC could affect local antituberculosis immune response and disease progression. With regard to *Mtb* infection, several immune modulatory properties of MSC are of interest, namely, their capacity to inhibit Th1 response (the main effector mechanism of anti-TB defense [36–39]), their ability to modulate macrophage and dendritic cell functions, and their ability to regulate exacerbated inflammatory reactions (an important factor of TB pathogenesis [25, 40]).

**Fig 2:**
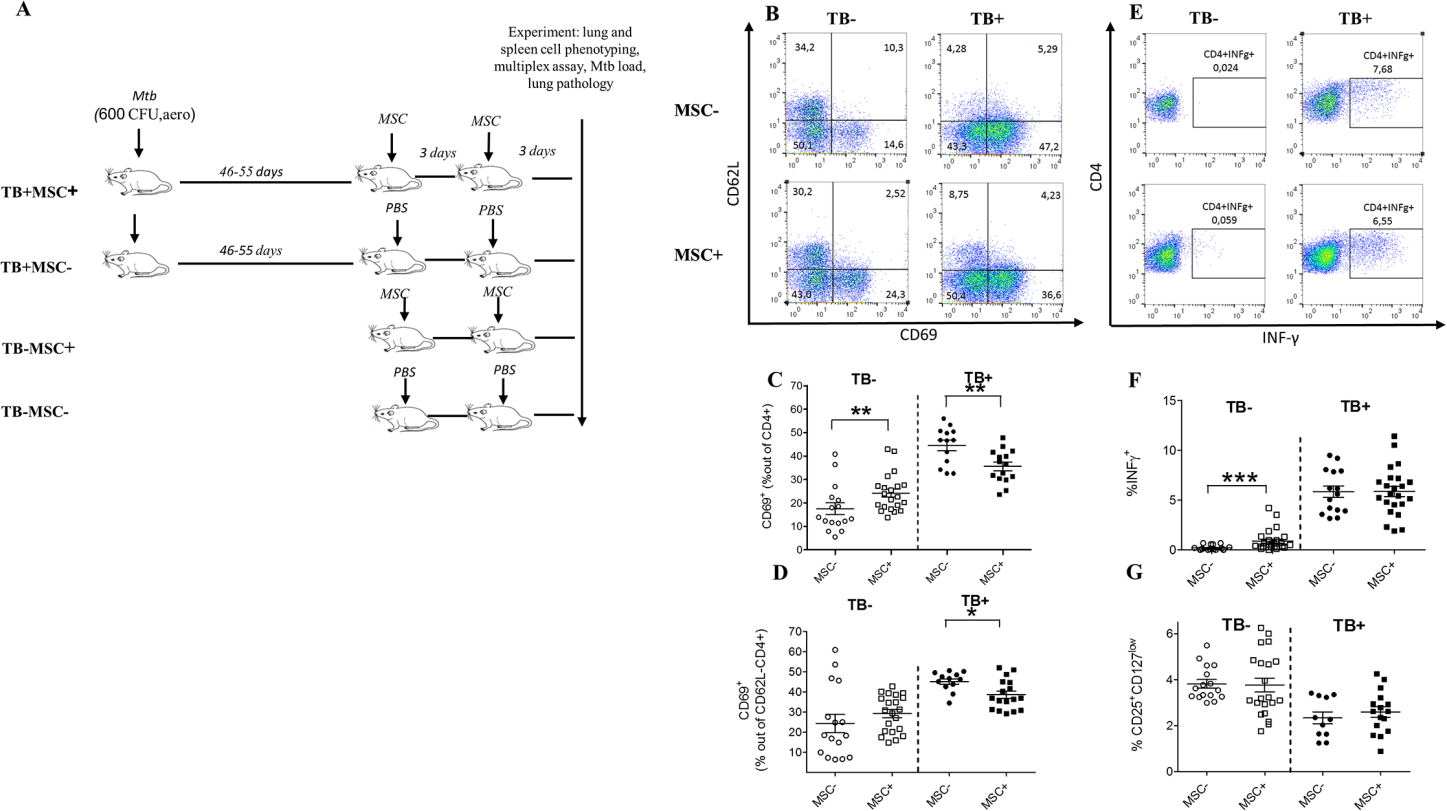
MSC stimulate lung CD4^+^ cell activation and IFN-γ production in uninfected mice and slightly suppress cell activation during *Mtb* infection. Uninfected mice and mice challenged with *Mtb* (600 CFU/mouse given aerogenically) were transferred with MSC (two injections with 3 days interval performed on days 46–55 post-challenge) or received injections of PBS. Three days after the last injection lung cell suspensions were analyzed for the expression of the indicated markers and IFN-γ. A, Scheme of experiments. B, Co-expression of CD62L and CD69 by lung CD4^+^ cells (representative example of flow cytometry data). C, D, summarized data showing percentages of CD69^+^ cells out of all CD4^+^ (C) or effector CD62L^-^CD4^+^ (D) lymphocytes. E, Identification of IFN-γ producing CD4^+^ cells in the lungs (representative example of flow cytometry data). F, G, Summarized data showing percentages of IFN-γ producing (F) and CD25^+^CD127^low^ (G) CD4^+^ cells. Data are summarized from 3 (*Mtb* infected mice) or 4 (uninfected mice) independent experiments (n = 11-19/group).

**Fig 3:**
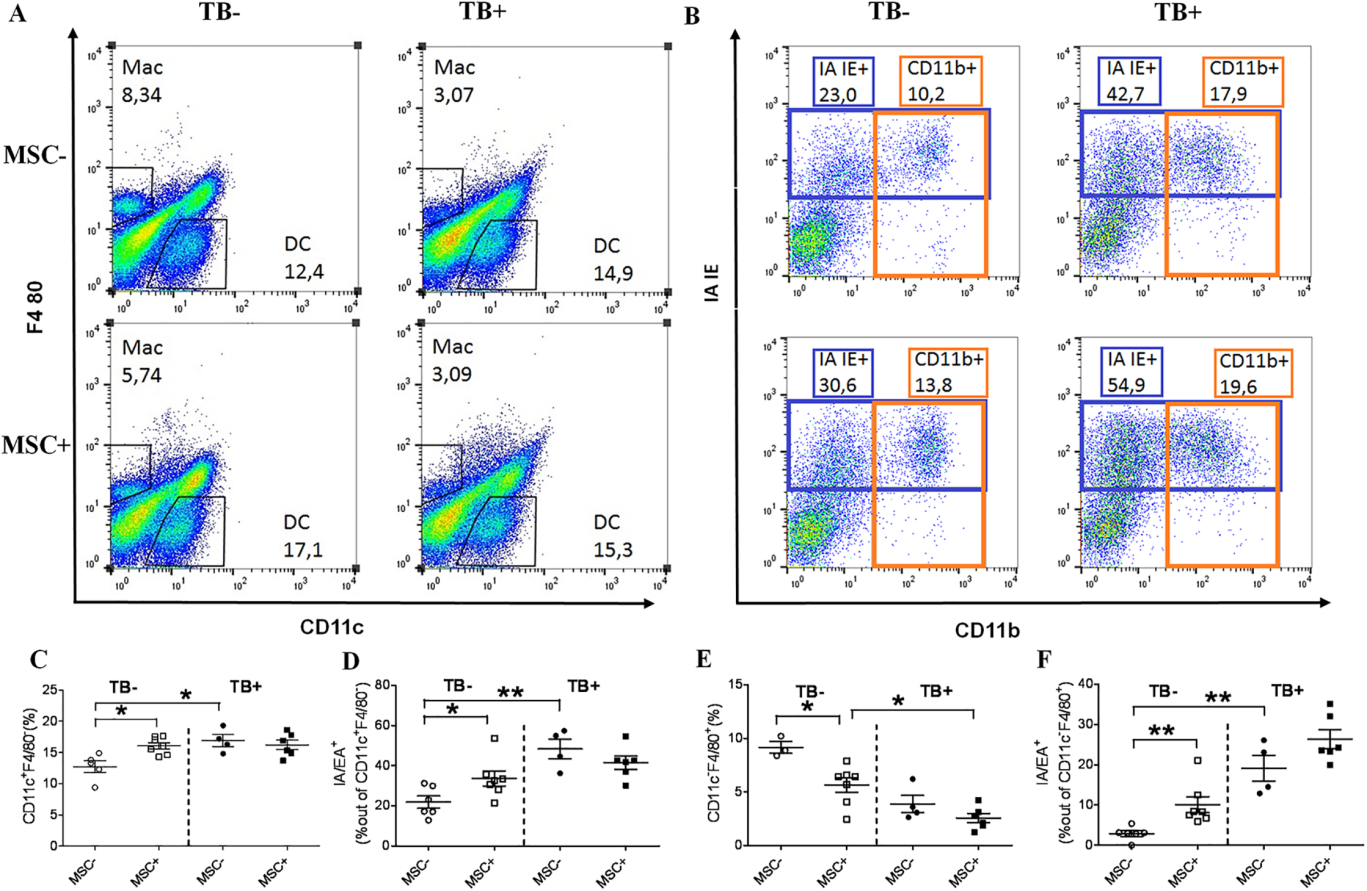
MSC promote the accumulation of DC and the activation of DC and macrophages in the lungs of uninfected mice. Uninfected mice and mice challenged with *Mtb* (600 CFU/mouse given aerogenically) were transferred with MSC as described in the legend to Fig 2 and lung cell suspensions were analyzed by flow cytometry. A, Identification of CD11c^+^F4/80^-^ (DC) and CD11c^-^F4/80^+^ (macrophages) populations by flow cytometry. B, Representative example showing the expression of IA/IE and CD11b by lung DC. C, E, Summarized data showing percentages of DC (C) and macrophages (E). D, F, Summarized data showing the expression of MHC class II by DC (D) and macrophages (F).

To address MSC ability to modulate these responses during *Mtb* infection, we challenged B6 mice with 600 *Mtb* CFUs aerogenically and transferred them with MSC (group TB^+^MSC^+^, Fig 2A). The cells were injected twice on days 46–55 post-challenge, i.e. at the stable stage of infection (according to our unpublished data, infection of B6 mice with 600 *Mtb* CFUs induced subchronic infection, mice survived for at least 100 days (time of observation)). Simultaneously with *Mtb* infected mice, MSC were transferred into uninfected animals (TB^-^MSC^+^ group). Two control groups, TB^+^ and TB^-^, were injected with PBS (TB^+^MSC^-^ and TB^-^MSC^-^, respectively). Three days after the second transfer, we analysed the percent of activated (CD69^+^), IFN-γ producing, IL-4 producing, and regulatory (CD25^+^CD127^low^/Foxp3^+^) CD4 T lymphocytes in the lungs and spleen of recipient mice (Fig 2). We also examined the percent and activation status of pulmonary macrophages (CD11c^-^F4/80^+^) and dendritic cells (CD11c^+^F4/80^-^, Fig 3).

In TB^+^MSC^-^ mice, lung CD4^+^ and effector CD4^+^CD62L^-^ lymphocytes were significantly more activated and contained higher percent of CD69^+^ cells (2,5 fold, p<0,0001) and IFN-γ producing cells (27,3 fold, p<0,0001) compared to TB^-^MSC^-^ mice (Fig 2C and 2F). Following MSC transfer to TB^+^ mice, the percentages of INF-γ producing cells remained unaltered (Fig 2F); the percentages of activated CD4^+^ and effector CD4^+^ T cells slightly decreased. Although the decrease was statistically significant (p<0.01 and p<0.05, respectively), it was moderate (1.3 and 1.2 fold, respectively, Fig 2C and 2D), questioning the biological significance of the observation. In contrast to their weak inhibitory effect on T cells during *Mtb* infection, in uninfected mice, MSC slightly promoted CD4^+^ cell activation (1.4 fold, p<0.01) and dramatically and highly reproducibly increased the percent of IFN-γ producing cells (5-fold, p<0.002, similar effects were observed in 5 independent experiments).

In both, TB^+^ and TB^-^ mice, MSC did not affect the activation status of CD4^+^ cells in the spleen (data not shown). IL-4 producing cells were rare in all groups of mice (<0.2% of CD4^+^ lymphocytes) and their frequency was not affected significantly by MSC transfer (data not shown).

Aside from effector T cells, MSC are known to stimulate the generation and function of regulatory T cells (Treg), which have potent antigen-specific and non-specific immune suppressive activity directed against different subpopulations of lymphoid and myeloid cells [11, 12]. Therefore, we tested whether MSC affected Treg population in our model. We found that the administration of MSC did not change the percentages of CD25^+^CD127^low^ or Foxp3^+^ cells in the lungs of TB^+^ or TB^-^ mice (Fig 2G and data not shown).

To address MSC effects on macrophages and DC, we determined the size of these populations and their expression of MHC class II molecules and CD11b integrin (Fig 3). Compared to TB^-^ mice, TB^+^ mice had higher frequency of DC (p<0.05, Fig 3C), higher percent of DC expressing MHC class II and CD11b (p<0.05 and p<0.01, respectively, Fig 3B and 3D and data not shown), and lower frequency of macrophages (p<0.02, Fig 3E) in their lungs. Administration of MSC did not affect macrophage and DC populations in TB^+^ mice (Fig 3). In contrast, the transfer of MSC to TB^-^ mice affected both populations, slightly increasing the frequency of DC (1,3 fold, p<0.05), decreasing the percent of macrophages (1,6 fold, p<0.05) and up-regulating the expression of MHC class II (by both populations, 1.6 and 3,5 fold, p<0.05 and p<0.01, respectively, Fig 3D and 3F) and CD11b (by DC, 2.0 fold, p<0.05, data not shown).

Overall, in the lungs of uninfected mice MSC exerted proinflammatory effect, increasing the pool of activated and IFN-γ producing CD4^+^ lymphocytes, augmenting the population of DC and elevating the activation status of DC and macrophages. However, the cells did not induce marked effects on cell populations residing in the lungs of *Mtb* infected mice.

### MSC modulate cytokine milieu in the lungs of uninfected mice

According to literature, MSC exert multiple modulatory effects on various immune cells aside from effector Th1 lymphocytes. We assumed that inside the lung tissue, this MSC-driven regulation of immune responses should be accompanied by changes in the production of cytokines and chemokines, associated with different types of immune responses. To track these effects, we evaluated the levels of 23 cytokines and chemokines in lung cell homogenates and plasma obtained from TB^+^MSC^+^, TB^+^MSC^-^, TB^-^MSC^+^ and TB^-^MSC^-^ mice. The analysis was performed using 23-plex assay that included the following list of cytokines: IL-1α, IL-1β, IL-2, IL-3, IL-4, IL-5, IL-6, IL-9, IL-10, IL-12(p40), IL-12-(p70), IL-13, IL-17, IFN-γ, KC (CXCL1), G-CSF, GM-CSF, MIP-1α (CCL3), MIP-1β (CCL4), MCP-1 (CCL2), TNF-α, RANTES (CCL5), eotaxin.

**Fig 4:**
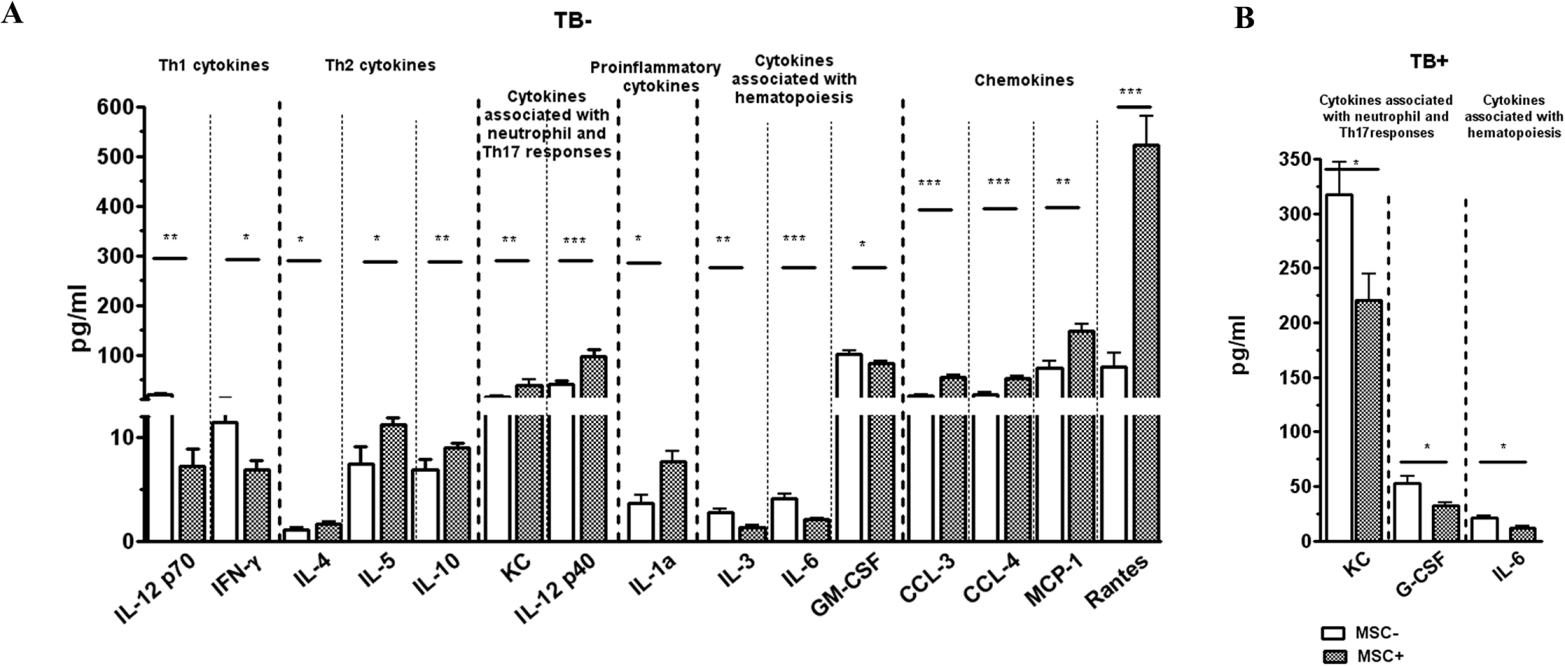
MSC effects on cytokine and chemokine levels in the lungs of uninfected and *Mtb* infected mice transferred with MSC or injected with PBS. Mice were infected and transferred with MSC as described in the legend to Fig 2. Cytokine and chemokine levels were determined in lung cell homogenates of uninfected (A) and *Mtb* infected mice (B) using 23-plex assay. Checked bars, mice transferred with MSC; open bars, mice injected with PBS. Only those factors that differed significantly between MSC transferred and control mice are shown (summarized data of 3 independent experiments, n = 8-14/group). For full list of data see S3 Fig.

The results were different in the lungs of *Mtb* infected and uninfected mice. In *Mtb* infected lungs, the levels of cytokines and chemokines were 2–4 (IL-9, TNF-α, GM-CSF, eotaxin) to more than 20 (IL-1β, IL-12p40, MIP-1α, RANTES, IL-1α) fold higher compared to uninfected lungs (Fig 4 and S2 Fig). Injection of MSC lowered the levels of IL-6 (1.7 fold, p<0.02), KC (1.4 fold, p<0.05), and G-CSF (1.7 fold, p<0.02) in the lungs of TB^+^ mice (Fig 4). There was also a tendency towards a lower expression of MCP (1.4 fold, p<0.1) and RANTES (1.5 fold, p<0.1) in TB^+^MSC^+^ compared to TB^+^MSC^-^ mice; other cytokines were not affected by MSC transfer (S2 Fig). Thus, in *Mtb* infected mice MSC slightly inhibited local production of factors associated with neutrophilic inflammation and hematopoiesis. Given that during *Mtb* infection neutrophilic inflammation may be involved in pulmonary pathology and TB progression [41–43], this effect could be beneficial for the host. However, analysis of cells expressing Gr-1, the marker of neutrophils, showed that MSC did not affect CD11b^+^Gr-1^hi^ (neutrophils) nor CD11b^+^Gr-1^dim^ (myeloid-derived suppressor cells, [27]) populations, at least in our model of stable TB infection (S3 Fig).

In the lungs of uninfected mice, MSC induced multiple effects (Fig 4 and S3 Fig). First, they decreased local production of cytokines associated with type 1 response (IL-12p70 and IFN-γ, 2.8 and 1.6 fold, p<0.02 and p<0,002, respectively) and hematopoiesis (IL-6, GM-CSF and IL-3, 2.0, 1.2 and 2.1 fold, p<0.0005, p<0.05 and p = 0.055, respectively). Second, MSC injection resulted in the increase in type 2 cytokines (IL-4, IL-5 and IL-10, 1.6 fold, 1.5 and 1.3 fold, p< 0.05, p<0.02, and p = 0.01, respectively). Third, MSC increased pro-inflammatory chemokines MIP-1α (3.1 fold, p<0.0005), MIP-1β (2.5 fold, p<0.0005), MCP (2.8 fold, p = 0.001), RANTES (6.9 fold, p<0.0005, Fig 4) and KC (2.5 fold, p<0.01). Fourth, MSC injection led to the increase in the amount of IL-12p40, molecule involved in the formation of IL-12 and IL-23, i.e. in type 1 and type 17 responses (2.43 fold, p<0.001). Overall, in the presence of MSC, the production of Th1 and hematopoietic factors was suppressed, whereas cytokines and chemokines associated with type 2 response, cell attraction and neutrophilic inflammation were overexpressed. The observed effects could not be simply attributed to the secretory activity of MSC, since changes in the level of cytokines in the lungs of MSC recipient mice differed from the pattern of cytokines observed in the supernatants of MCS cultures. For example, MCP-1, KC, IL-6 and eotaxin were the predominant cytokines in the supernatants of MSC cultures (>100 pg/ml, S4 Fig), whereas following the transfer of MSC to uninfected mice, the levels of KC and MCP-1 only slightly increased, IL-6 was depressed, and eotaxin was not affected (Fig 4 and S4 Fig).

In contrast to the lungs, in plasma MSC injection did not induce significant changes in cytokine profile (S3 Fig).

### In the lungs of recipient mice, MSC locate predominantly in the alveolar walls and are absent from the inflammatory foci

**Fig 5:**
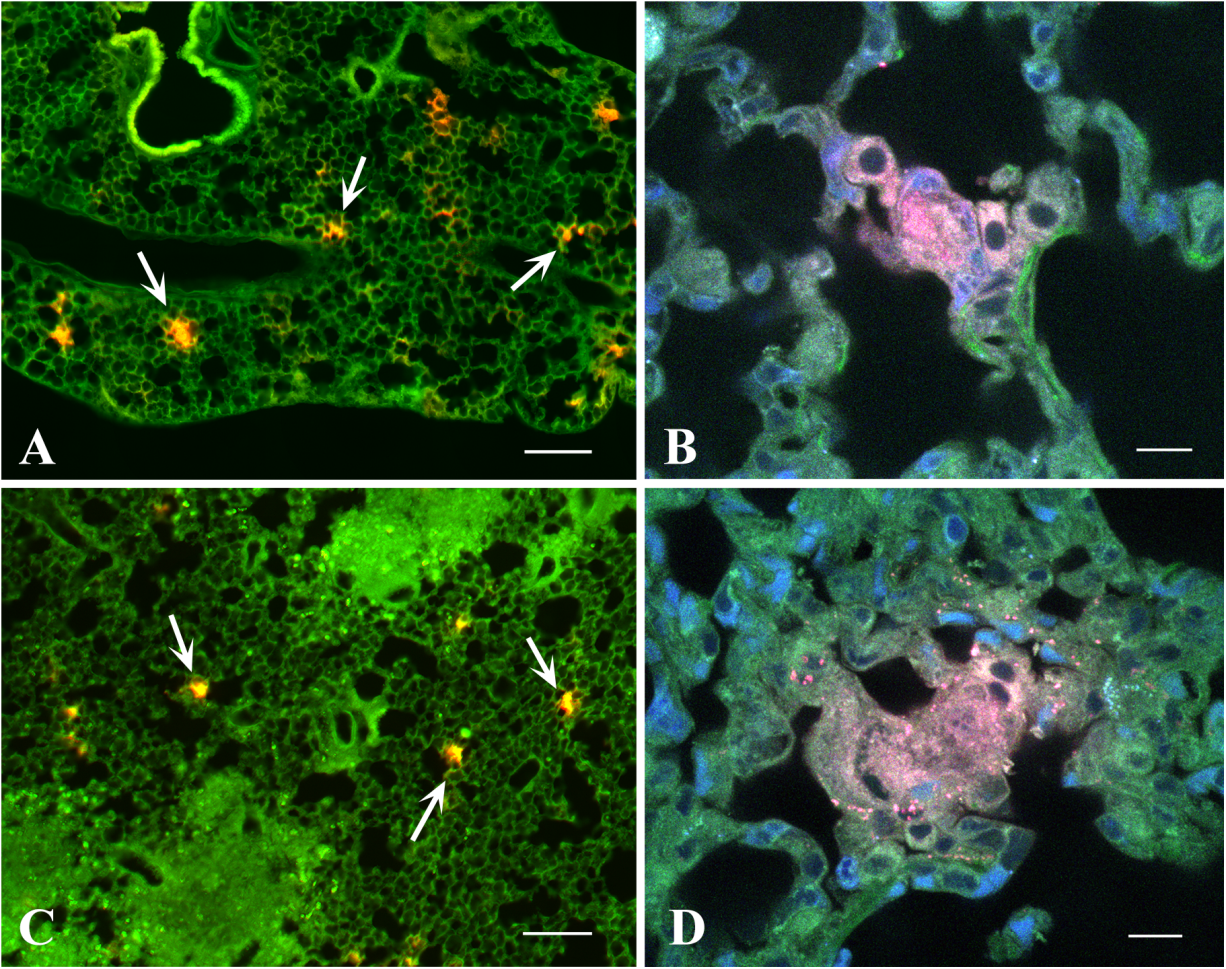
**Localization of MSC in lung tissue of uninfected (A, B) and *Mtb* infected TB**^**+**^ **(C, D) mice, 1 h after the transference.** MSC were labeled with PKH26 (red), and the slices were counterstained with ActinGreen 488 (green) and Hoechst 33342 (blue). A, C, An overview showing MSC in the tissue (arrows), epifluorescence images were obtained by automatic stitching of 36 intermediate images (Keyence Image Joint Function). B, D, High magnification confocal images of MSC aggregations (x63, each image is a maximum intensity projection of 5x0.6 μm optical slices). Scale bars: A, C = 200 μm; B, D = 10 μm. Similar MSC localization was observed 1 and 2 days after the transfer.

Our data showed that MSC transfer dramatically changed immune cell functions and cytokine/chemokine milieu in the lungs of control mice, particularly, increasing local production of proinflammatory chemokines mediating immune cell migration. In contrast, in *Mtb* infected mice, MSC did not affect significantly pulmonary immune reactions. Proinflammatory effects of MSC observed in uninfected mice could be attributed to the immunomodulatory properties of MSC or to their trapping to lung capillaries leading to the capillary blockade and subsequent inflammation. In turn, lack of MSC effects during *Mtb* infection could be explained by cell inability to modulate a high-level background inflammation or to poor accessibility of inflammatory foci for MSC. To address these possibilities, we next examined the localization of MSC in the lungs of recipient mice (Fig 5).

MSC were labeled with PKH26 and transferred i.v. to uninfected and *Mtb* infected recipient mice. Lung sections were examined one hour and 1–2 days post-transfer. In uninfected mice MSC were found as cell aggregates in the alveolar walls (Fig 5A and 5B). Unexpectedly, in *Mtb* infected mice, MSC had similar localization: their aggregates were found in the alveolar walls of the relatively unaffected areas of the lungs, whereas the cells were absent from the inflammatory foci where alveolar architectonics was disrupted (Fig 5C and 5D). The results indicated that MSC were, indeed, trapped in the pulmonary capillar bed, and suggested that the capillary blockade could cause proinflammatory effects of MSC observed in our study.

To further check this hypothesis, we decided to examine whether unrelated cultured large cells would cause similar effect. We transferred uninfected mice with NIH/3T3 fibroblast cells and analyzed cytokine/chemokine expression in lung lysates. As shown in S5 Fig, fibroblast transfer increased the levels of only two factors, RANTES and IL-12p70, and only to a small degree (1.9 and 1.3-fold, respectively, p<0.05; note that MSC transfer elevated the levels of these cytokines 3.1 and 6.9 fold and also affected other factors, Fig 4). Thus, in our study proinflammatory effect was largely restricted to MSC, suggestively due to their high adhesivity leading to capillary blockade.

### MSC injections fail to affect the course of *Mtb* infection

**Fig 6:**
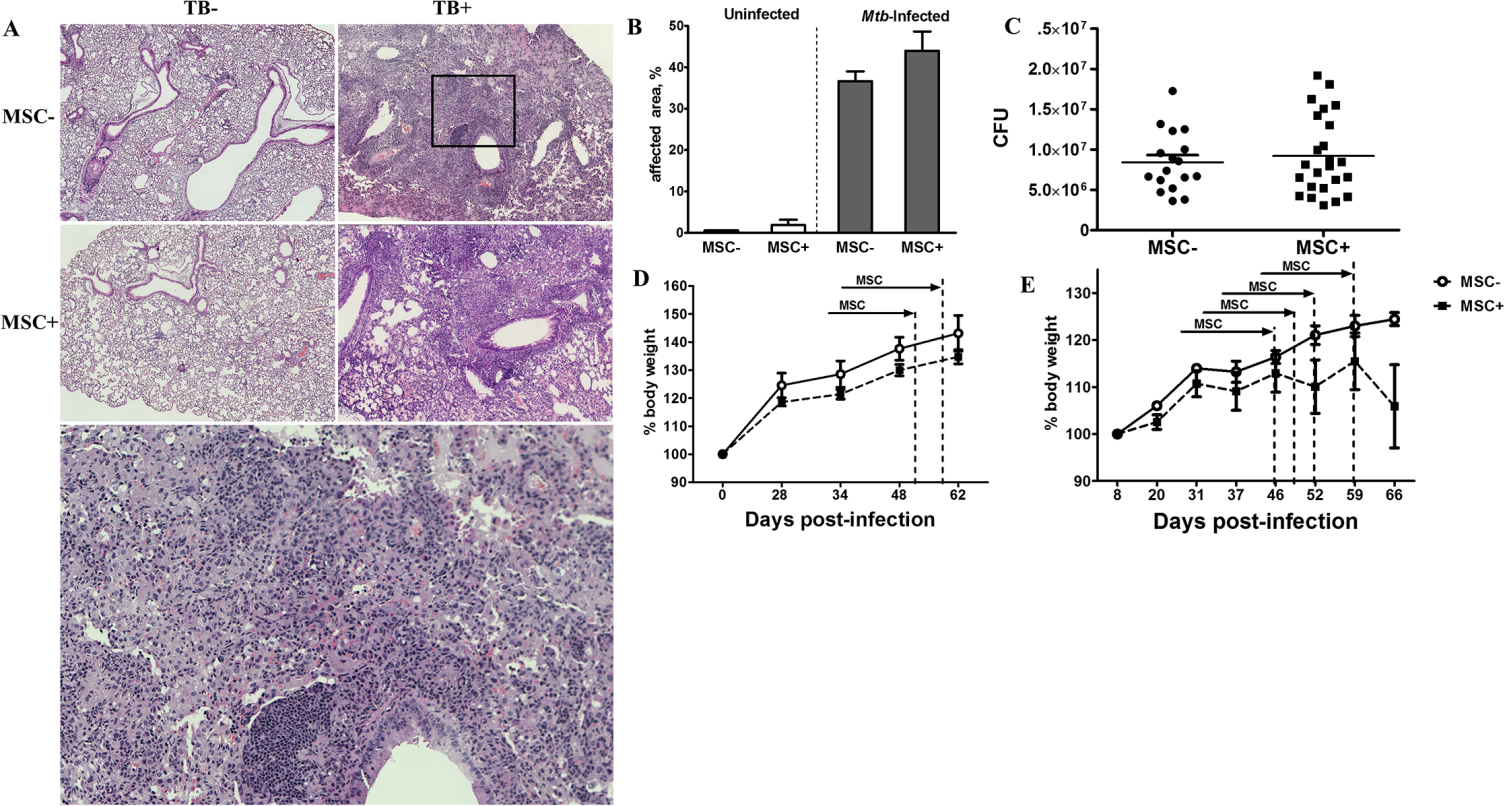
MSC transfer does not affect the course of *Mtb* infection in mice. A-D. Mice were challenged with *Mtb* (600 CFUs/mouse), transferred with MSC or PBS (twice) and analyzed at 3 days after the last transfer (see legend to Fig 2). A, Representative sections of HE staining of lungs from TB^-^ and TB^+^ mice injected with MSC (MSC^+^) or PBS (MSC^-^, magnification x65) and the inset showing higher-magnification view of the inflammatory focus from *Mtb* infected mouse. B, Area (percent) of lung tissue affected by inflammation (mean + SE). C, *Mtb* load in the lungs of TB^+^ mice injected with MSC or PBS. D, Weight dynamics (100%—weight on the day of *Mtb* challenge; the results of one representative out of 3 independent experiments are shown). E, Mice challenged with *Mtb* received 4 MSC transfers. Weight dynamics is shown (one experiment). All differences between the groups were insignificant.

Several recent studies suggested that MSC can potentially be used as an adjunct therapy for TB treatment to improve clinical outcome of severe TB [19–21]. Our data on local immune modulatory effects of MSC in the lungs suggested that the cells could influence the disease course by attenuating local inflammation. Therefore, we assessed whether MSC influenced lung *Mtb* burden and disease progression. The later was assessed by determining the degree of pulmonary pathology and body weight loss. We found that MSC did not decrease *Mtb* burden or pulmonary pathology, and did not improve body weight dynamics (Fig 6A–6D). Lack of effect could be due to the rapid elimination of MSC from the pulmonary tissue. To prolong the potential effect of MSC, MSC were transferred to *Mtb*-challenged mice four times (starting day 46 post *Mtb* challenge with 6–10 days intervals between injections). *Mtb* burden was still unaffected and no significant improvement of body weight was found in TB^+^MSC^+^ compared to TB^+^MSC^-^ mice (Fig 6E).

Thus, MSC ability to slightly modulate local immune responses in the lungs of *Mtb* infected mice did not lead to any beneficial effect in terms of disease pathology and progression.

## Discussion

MSC acquired a reputation of cell population which is readily accessible, stable and suitable for the goals of regenerative medicine. These multipotent cells have been studied well enough to prove their ability to differentiate *in vitro* into several cell types (i.e., adipocytes, chondrocytes and osteocytes). Besides that, MSC secrete impressive set of soluble factors suggesting that upon transplantation, the cells may affect tissue repair, proliferation and differentiation of different cell types in paracrine manner. A related property of MSC is their immunomodulatory activity, giving a hope that MSC transplantation could be used to treat undesired inflammation and tissue damage in the course of pathological immune responses. However, MSC properties depend on cell environment. Thus, MSC use as a clinical tool requires better knowledge of their trafficking inside the body and their effects on immune system under particular pathological conditions. Recently several authors suggested using MSC transplantation as an adjunct therapy for multi-drug resistant TB [19–21]. However, immunomodulatory properties of MSC in *Mtb* infected tissues remain largely unknown.

In this study we have tested: (i) dynamics, tissue distribution and localization in the lungs of MSC derived from adipose tissue and transferred to *Mtb* infected and uninfected mice; (ii) local immunological effects of MSC in the lungs and their systemic effects during *Mtb* infection and under steady-state conditions.

We show that MSC tissue distribution depends on the route of their injection. Intraperitoneal administration led to short term accumulation of MSC in liver and spleen. Following systemic (i.v.) transfer, MSC accumulated preferentially in the lungs, where they were most numerous during the first 3 days post-transfer and declined by day 10. The data correspond well to the results of other studies [34, 44, 45]. Recently it was even suggested to use MSC capacity to accumulate in the lungs for the delivery of certain agents into the lung tissue [35, 45]. However, MSC’s accumulation in the lungs does not necessarily mean that cells had migrated to lung parenchyma. Other possibilities are that CFSE^+^ cells identified in lung tissue suspensions represent injected cells circulating in the bloodstream or attached to lung capillary wall. Several arguments support the latter explanation. First, we carefully washed out lung vessels, which makes the possibility of detecting circulating cells unlikely. Second, the percentages of CFSE^+^ cells in the lungs were significantly higher compared to blood at all time-points (including day 0), which further rules out blood cell impact on the data. Finally, our analysis of lung sections directly identified aggregates of MSC in alveolar walls, supporting the hypothesis of cell localization in the capillary and high probability of MSC-mediated capillary blockade. Thus, we believe that CFSE^+^ cells found in the lungs represent non-circulating MSC attached to lung capillary wall. This concept also explains rapid increase in CFSE^+^ cells in the lungs (by 3 h post-infection) and their higher percent in the lungs compared to blood. The result is also in line with other reports that suggested that trapping of i.v. transferred MSC occurs in the first microcapillary network they encounter [46]. MSC trapping in lung capillary raises a question about potential limitations of systemic MSC infusion, particularly, in patients with lung pathologies, and suggests that the safety of the approach should be carefully examined.

The fact that the majority of infused MSC were trapped in the lungs raised a question about their local pro- and/or anti-inflammatory effects. These effects have not been analyzed in details so far. Given that immunomodulatory properties of MSC critically depend on cell environment, we have used two different experimental approaches. The first, to address the effects of MSC under steady-state conditions; and the second, to define MSC functions in the course of experimental *Mtb*-infection in the lungs.

In line with other studies, we observed that *Mtb* infection induced significant pulmonary inflammation and local immune responses. This was documented by a higher content of activated (CD69^+^) and IFN-γ producing lymphocytes and a higher local level of pro-inflammatory cytokines and chemokines in *Mtb* infected compared to uninfected lungs (Figs 2, 3 and 4). Type 1 and proinflammatory conditions are known to activate MSC suppressive properties [3, 4, 13–15]. However, in our study, in the settings of *Mtb* infection, MSC immunosuppressive activity was poor and limited to a weak inhibition of CD4^+^ cell activation (1.3 fold) and a modest suppression of factors associated with neutrophilic inflammation and hematopoiesis (KC, G-CSF, IL-6, 1.4–1.7 fold). Because the effects were slight, their biological significance is questionable. Indeed, in line with their poor immunological activity in *Mtb* infected mice, in our hands MSC did not exert beneficial effect on the course of *Mtb* infection, i.e., they did not affect *Mtb* burden, pulmonary pathology or disease progression. The data seem to contradict the results by Skrahin and co-authors [19, 21] who reported a therapeutic effect of MSC in patients with MDR-TB [19,21]). The discrepancies between the studies can be attributed to the differences in their settings, including the differences in TB pathogenesis in mice and human. Indeed, Skrahin and co-authors [19, 21] analyzed MSC effects in patients receiving anti-tuberculosis drugs and MSC as adjunct therapy [19, 21]. We explored MSC effects using an experimental model of TB infection in mice not receiving chemotherapy. Lack of beneficial effects of MSC on the course of *Mtb* infection observed in our study is in a good agreement with a proposed short-range paracrine activity of MSC and our data showing preferential localization of MSC outside of the inflammatory foci. Of note, MSC ability to reduce inflammation and inflammation-associated lung damage has previously been shown almost exclusively in the models of acute lung injury and diffuse inflammation [20, 35, 47].

According to our data, in the absence of *Mtb* infection, MSC exerted significant pro-inflammatory action in the lungs. In particular, their infusion induced up-regulation of factors attracting neutrophils, lymphocytes and monocytes (MIP-1α, MIP-1β, MCP-1, RANTES, KC) and promoted the accumulation of activated and IFN-γ producing cells in the lung tissue. To the best of our knowledge, these effects have not been reported previously. This is likely due to the fact that the majority of studies focused on MSC effects outside the lungs. In our study, we either did not register significant modulation of cytokine/chemokine profile in the blood or the accumulation of CD69^+^ and IFN-γ^+^ CD4^+^ cells in spleen and other organs of mice transferred with MSC. Thus, only local, lung tissue restricted proinflammatory effects of i.v. transferred MSC were observed under steady-state conditions. As discussed above, these effects likely result from MSC clogging of pulmonary capillary. Further investigations are needed to explore the mechanisms of MSC proinflammatory activity in the lungs in more details and to understand how these effects may affect potential MSC use.

Besides proinflammatory effects, MSC modulated the type of immune reactivity in the lungs, i.e. they dampened the levels of IFN-γ and increased the levels of IL-4, IL-5 and IL-10. This is consistent with the effects described in other studies where MSC were shown to bias immune reactivity from type 1 to type 2 [2–4]. Our data on MSC-driven inhibition of IFN-γ levels and stimulation of type 2 cytokines in the lungs of uninfected mice seem to contradict increased percentages of IFN-γ^+^ and unaltered low frequencies of IL-4^+^ cells in the lungs of the same mice. The discrepancy may be attributed to the differences between the experimental settings that we used to measure cytokine levels and identify cytokine producing cells. Specifically, cytokine levels were measured in lung homogenates *ex vivo* without cell re-stimulation. In contrast, IFN-γ and IL-4 producing cells were identified *in vitro* following cell re-stimulation. One could expect that MSC located in the lungs can dampen cytokine production by local Th1 cells and stimulate the production of IL-4 by Th2 and other lung cells leading to a decrease in IFN-γ and an increase in IL-4 levels in lung homogenates. In contrast, in *in vitro* cultures, MSC-T cell interactions were lost, which could result in the restoration of T cell reactivity. In line with this explanation, several studies have demonstrated that MSC-mediated suppression of IFN-γ production is reversible upon MSC withdraw [48, 49].

Overall, in this study we show that: (i) following systemic administration, MSC migrate predominantly to the lungs, where they can be identified until day 10 post-transfer; (ii) in the lung tissue, transferred MSC localize in the alveolar septa as cell aggregates and do not penetrate to the inflammatory foci; (iii) local pulmonary effects of MSC differ under steady-state and inflammatory conditions; (iv) under steady-state conditions, MSC exert significant pro-inflammatory effects in the lungs that manifest as an increase in the percent of IFN-γ producing lymphocytes and dendritic cells and local production of chemokines mediating immune cell migration and inflammation; (v) during *Mtb* infection, MSC fail to significantly modulate local immune responses and the course of disease. Thus, MSC administration can dramatically change T cell function and cytokine/chemokine milieu in the lungs, indicating that MSC effects and safety should be evaluated locally at the site of cell predominant post-transfer localization. Besides that, the data argue against the strong therapeutic potential of MSC during active TB.

## Supporting information

S1 Fig MSC inhibit activation of CD3-stimulated purified CD4+ cells in mixed cultures. T cells were isolated from uninfected (TB-) or Mtb infected (TB+) mice, stimulated with anti-CD3 antibodies (α-CD3+) or left unstimulated (α-CD3-), and cultured with MSC (10:1 ratio). Surface expression of CD62L and CD69 by CD4+ cells was determined 48h later. A, Percent of CD62L+ cells; B, absolute numbers of CD62L+ cells; C, percent of CD69+ cells; D, absolute numbers of CD69+ cells. Data of three independent experiments are presented.
(TIF)

S2 Fig **Cytokine and chemokine levels in the lungs (A, B) and plasma (C, D) of mice transferred with MSC. A-D,** Uninfected and *Mtb* infected mice were transferred with MSC as described in the legend to Fig 2. Three days after the last MSC transfer, lungs and plasma were collected. Cytokine and chemokine levels were determined in lung cell homogenates (A, B) and plasma (C, D) of uninfected (A, C) and *Mtb* infected (B, D) mice using 23-plex assay. Checked bars, mice transferred with MSC, open bars, mice injected with PBS. Data are summarized from 3 independent experiments (n = 8-14/group).
(TIF)

S3 Fig MSC transfer does not affect the percentages of CD11b+Gr-1hi and CD11b+Gr-1dim cells in the lungs. Mice were challenged with Mtb and transferred with MSC as described in the legend to Fig 2. The cells were examined 3 days after the last MSC transfer.
(TIF)

S4 Fig Cytokine and chemokine levels in the supernatants of MSC cultures. Supernatants were collected from MSC cultures at passages 3–4. Summarized data of 5 independent experiments are shown.
(TIF)

S5 Fig Transfer of fibroblast cells does not change significantly the cytokine and chemokine levels in the lungs of recipient mice. Uninfected mice were transferred with NIH/3T3 fibroblast cells according to the protocol used for the transfer of MSC. Cytokine and chemokine levels were determined in lung cell homogenates (A) and blood (B) 3 days after the last transfer using 23-plex assay. Checked bars, mice transferred with fibroblasts, open bars, mice injected with PBS (n = 7-12/group, 2 independent experiments).
(TIF)

## Abbreviations

i.v.: intraveneous
i.p.: intraperitoneal
MSC: mesenchymal stromal cells
*Mtb*: *Mycobaterium tuberculosis*
TB: tuberculosis
