# Supplementary figures and images for: The introduction of mesenchymal stromal cells induces different immunological responses in the lungs of healthy and M. tuberculosis infected mice
Source: PLoS One. 2017 Jun 8;12(6):e0178983. doi: 10.1371/journal.pone.0178983 (PMC5464766; doi:10.1371/journal.pone.0178983)

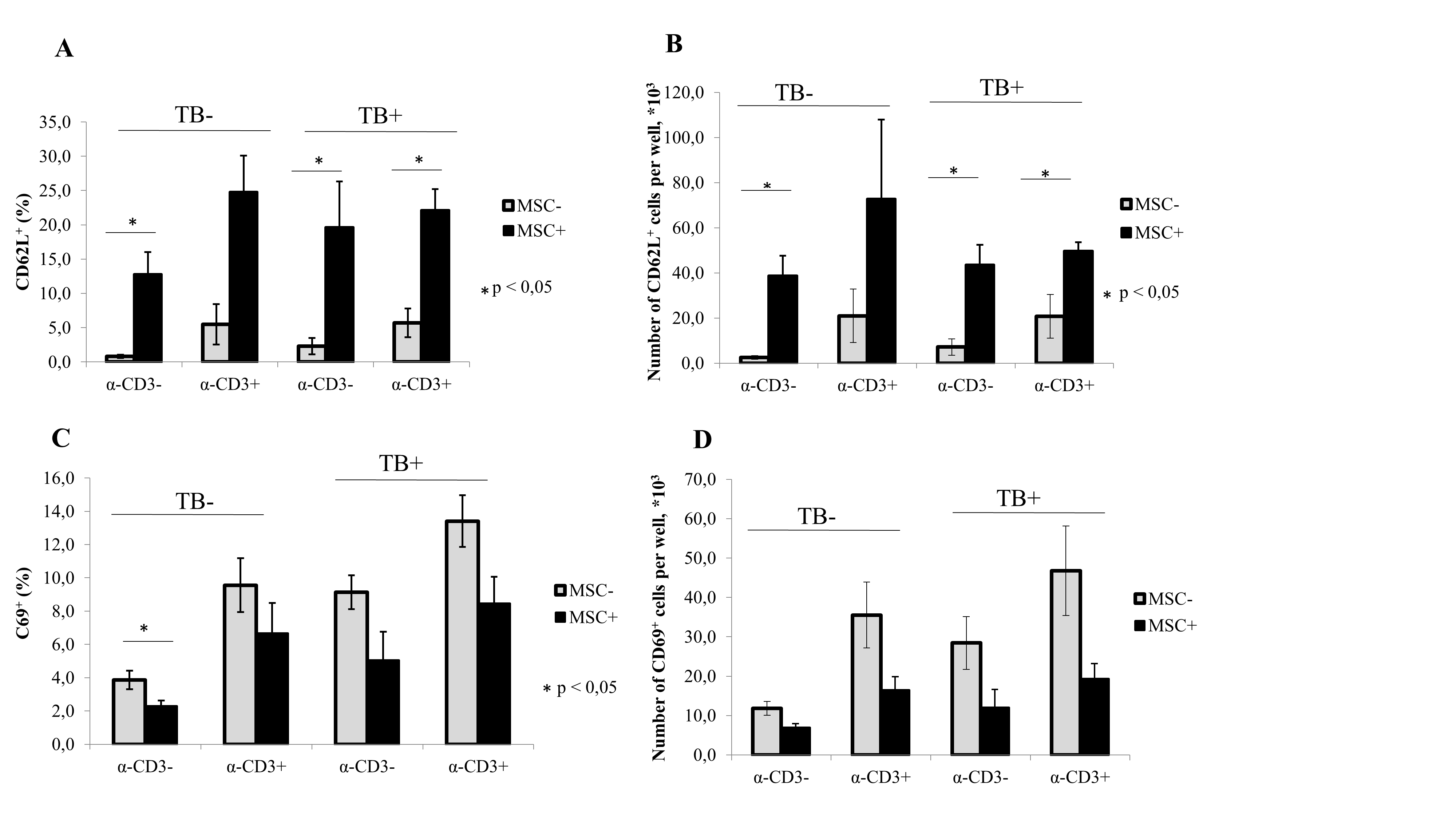

Supplement: S1 Fig — T cells were isolated from uninfected (TB-) or Mtb infected (TB+) mice, stimulated with anti-CD3 antibodies (α-CD3+) or left unstimulated (α-CD3-), and cultured with MSC (10:1 ratio). Surface expression of CD62L and CD69 by CD4+ cells was determined 48h later. A, Percent of CD62L+ cells; B, absolute numbers of CD62L+ cells; C, percent of CD69+ cells; D, absolute numbers of CD69+ cells. Data of three independent experiments are presented. (TIF) [file pone.0178983.s001.tif]

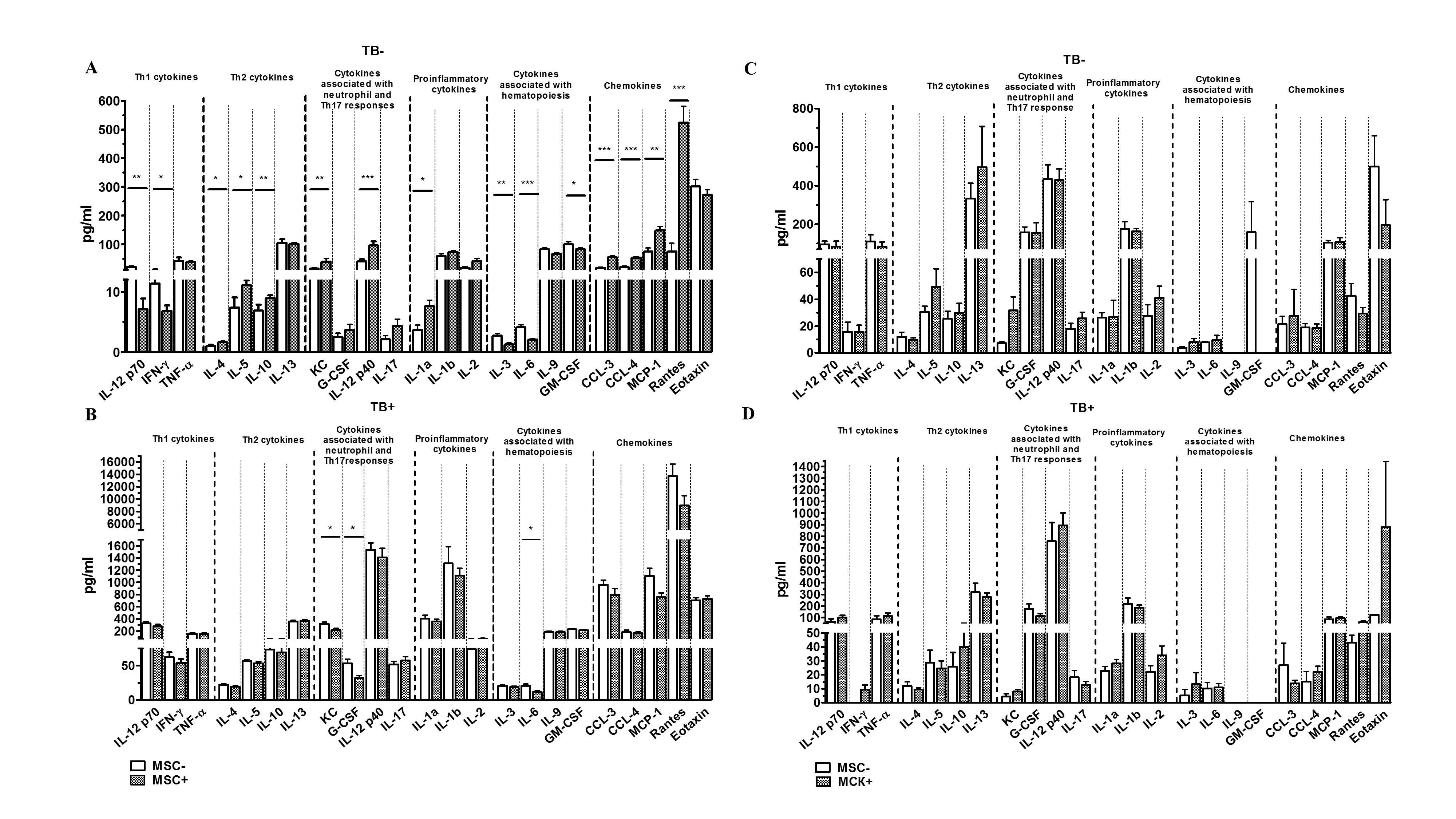

Supplement: S2 Fig — Cytokine and chemokine levels in the lungs (A, B) and plasma (C, D) of mice transferred with MSC. A-D, Uninfected and Mtb infected mice were transferred with MSC as described in the legend to Fig 2. Three days after the last MSC transfer, lungs and plasma were collected. Cytokine and chemokine levels were determined in lung cell homogenates (A, B) and plasma (C, D) of uninfected (A, C) and Mtb infected (B, D) mice using 23-plex assay. Checked bars, mice transferred with MSC, open bars, mice injected with PBS. Data are summarized from 3 independent experiments (n = 8-14/group). (TIF) [file pone.0178983.s002.tif]

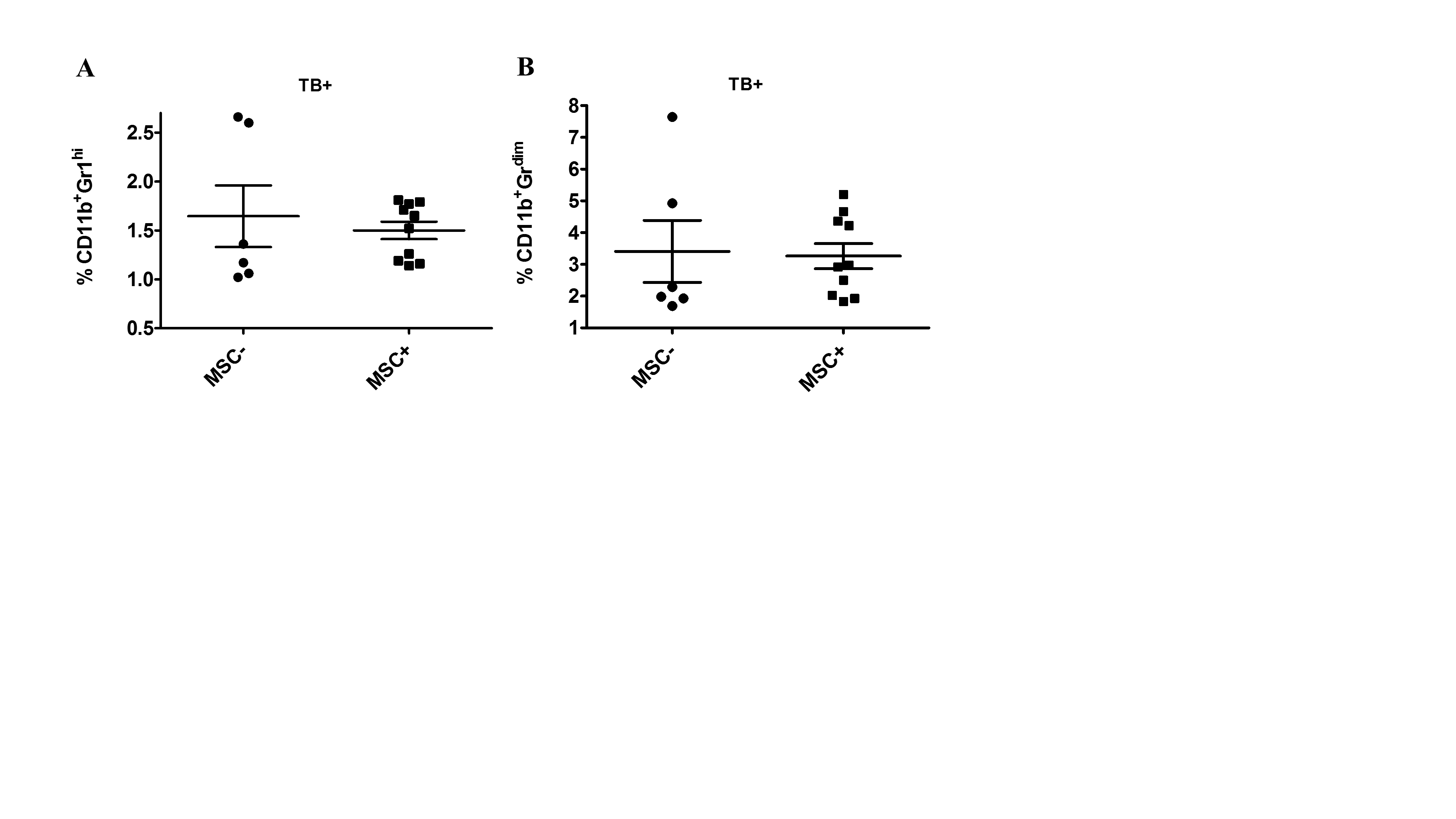

Supplement: S3 Fig — Mice were challenged with Mtb and transferred with MSC as described in the legend to Fig 2. The cells were examined 3 days after the last MSC transfer. (TIF) [file pone.0178983.s003.TIF]

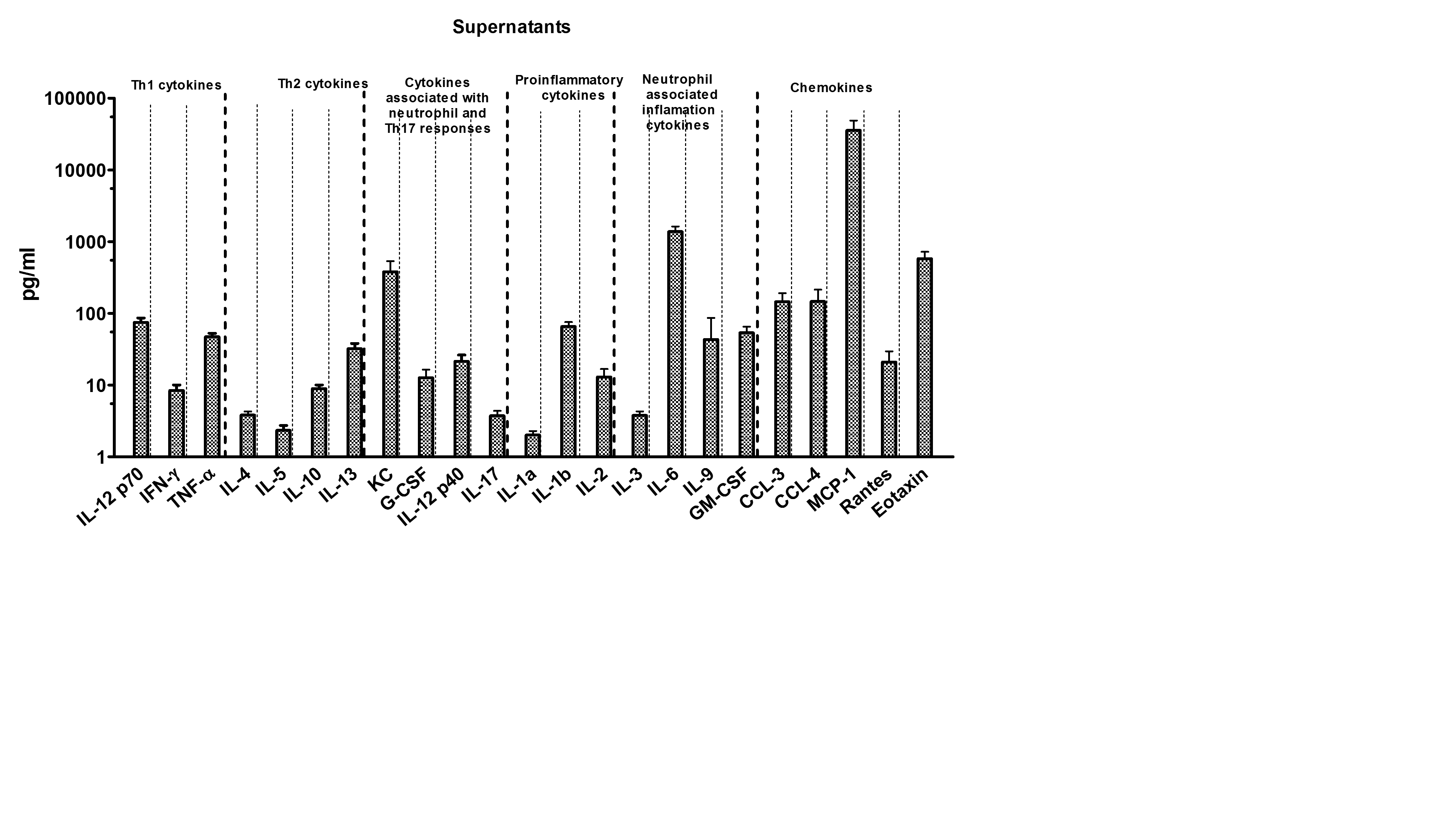

Supplement: S4 Fig — Supernatants were collected from MSC cultures at passages 3–4. Summarized data of 5 independent experiments are shown. (TIF) [file pone.0178983.s004.TIF]

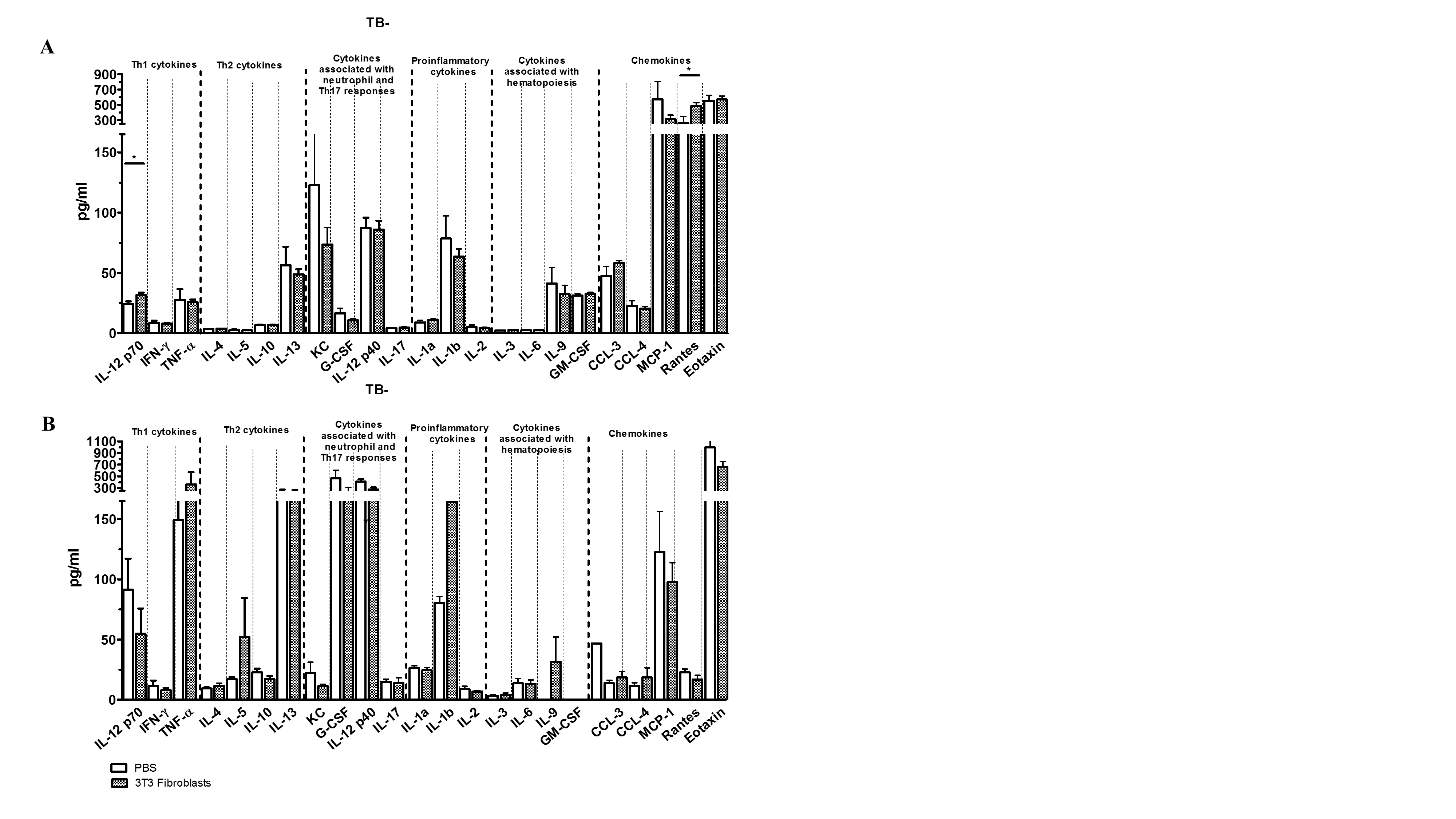

Supplement: S5 Fig — Uninfected mice were transferred with NIH/3T3 fibroblast cells according to the protocol used for the transfer of MSC. Cytokine and chemokine levels were determined in lung cell homogenates (A) and blood (B) 3 days after the last transfer using 23-plex assay. Checked bars, mice transferred with fibroblasts, open bars, mice injected with PBS (n = 7-12/group, 2 independent experiments). (TIF) [file pone.0178983.s005.TIF]
